# 

**DOI:** 10.1038/sj.bjc.6600378

**Published:** 2002-01-02


cell culture conditions (ie MET+HCY- [the symbols + and – denote the presence or absence of supplements in the media respectively]), HTC cells grew with a doubling time of 20 h in the exponential phase. In MET-HCY+ media, no cell growth occurred and cells accumulated in the S/G2 phase of the cell cycle. The cells remained viable during the period of MET deprivation as replacement of MET-HCY+ media with MET+HCY-media (after 5 and 10 days of MET-HCY+ treatment) resulted in cell proliferation and a normalisation of cell cycle parameters 3 to 4 days later. Both Phi-1 and Hs-27 cells (MET independent) were able to grow in MET-HCY+ media albeit at a reduced rate compared with cells in MET+HCY- media (doubling time for Hs-27 cells in MET+HCY- or MET-HCY+ media were 24 and 72 h respectively). Under MET-HCY+ conditions, no significant changes in cell cycle parameters were observed for Phi-1 and Hs-27 cells compared with parameters obtained in MET+HCY- media. This study suggests that the differences that exist between tumour and normal cells in terms of their MET dependence may provide a novel biochemical strategy by which MET dependent cells could be synchronised into specific phases of the cell cycle. As the cell cycle parameters of MET independent cells (ie normal cells) are not altered under MET deprived conditions, the appropriate use of cell cycle phase specific agents may therefore improve the therapeutic index of both standard chemotherapeutic agents and novel therapeutic agents. This work was supported by Cancer Research UK.

**P164**

**PROTEIN DEGRADATION IN SKELETAL MUSCLE IS INDUCED BY A PROTEOLYSIS INDUCING FACTOR (PIF) AND IS ASSOCIATED WITH INCREASED EXPRESSION OF MEMBERS OF THE UBIQUITIN-PROTEASOME PATHWAY PROTEOLYSIS-INDUCING FACTOR (PIF) AND WITH THE ACTIVATION OF THE TRANSCRIPTION FACTOR NFκB.**

AS Whitehouse* and MJ Tisdale, Pharmaceutical Sciences Research Institute, Aston University, Birmingham, B4 7ET, UK

Proteolysis Inducing Factor (PIF), isolated from a cachexia-inducing murine tumour, has been shown to stimulate protein catabolism in C2C12 myotubes. Protein degradation was enhanced by PIF after 24hours, which corresponded with an elevation in the chymotrypsin-like enzyme activity of the proteasome (the dominant catalytic activity of the β-subunits) and expression of 20Sα -subunits at concentrations of PIF between 2 and 16nM. Higher concentrations of PIF had no effect. A similar pattern was seen for expression of subunits of the 19S regulatory particle and $E2_{14k}$ (the ubiquitin conjugating enzyme). The action of PIF was attenuated by the polyunsaturated fatty acid - eicosapentaenoic acid (EPA) (50μM). At a concentration of 4nM, PIF induced a transient decrease in IκBα levels after 30min incubation, while no effect was seen at 40nM PIF. The level of IκBα, an NFκB inhibitory protein, returned to normal after 60min. Depletion of IκBα fr! om the cytosol was not seen in myotubes pre-treated with EPA, suggesting that the NFκB / IκB complex was stabilised. At concentration of 2 and 8nM, PIF stimulated an increased nuclear migration of NFκB, which was not seen in myotubes pre-treated with EPA The PIF-induced increase in chymotrypsin-like enzyme activity was attenuated by the NFkB inhibitor peptide -SN50, suggesting that NFκB may be involved in the PIF-induced increase in proteasome expression. The results further suggest that PIF co-ordinately upregulates expression of members of the ubiquitin -proteasome proteolytic pathway and that EPA may attenuate protein catabolism, at least partly, by preventing NFκB accumulation in the nucleus.

**P165**

**QUANTITATIVE ANALYSIS OF THE AH RECEPTOR SIGNALLING PATHWAY.**

Morag C.E. McFadyen*, Patrick H Rooney, Graeme I. Murray. Department of Pathology, University of Aberdeen, Aberdeen, AB25 2ZD UK.

The aryl hydrocarbon receptor (AhR) is a ligand activated receptor which on dimerisation with the aryl hydrocarbon nuclear translocator (ARNT) binds to xenobiotic responsive elements (XREs) that promotes the activation of a battery of genes, including the cytochrome P450 drug metabolising enzymes CYP1A1 and CYP1B1. These two P450s are known to demonstrate distinct cell type expression. Indeed, we have previously shown that CYP1B1 is a tumour-specific P450[1] and recently established several anti-cancer drugs as substrates for CYP1B1[2]. The aim of this study was to investigate the mechanism of cellular regulation of CYP1A1 and CYP1B1 by real time quantitative RT-PCR in three cell lines (MCF7, HEPG2 and MOG-G-CCM) known to differentially express CYP1A1 and CYP1B1 mRNA. In this investigation basal and inducible levels of CYP1A1 and CYP1B1 mRNA were determined for the three cell lines. The cells were exposed to the Ah receptor agonist 3-methylcholanthrene (3-MC) for a 12hr time period to determine the levels of inducible CYP1A1 and CYP1B1 mRNA. Specific primers and fluorescently labelled probes were designed for each cDNA, quantification of the mRNA was determined by the ABI7700 sequence detection system. Detection of the AhR and ARNT transcripts demonstrated basal levels for each amplicon, minimal changes in expression levels was observed on exposure to 3-MC. However, our results demonstrated differential basal and inducible expression of CYP1A1 and CYP1B1 in the cell lines. CYP1A1 basal expression was 3 and 5 fold higher in the HEPG2 cells compared to the MCF7 and MOG-G-CCM cells respectively. Where as basal CYP1B1 expression in the MCF7 and MOG-G- CCM cells was 500 and 4,000 fold higher than in the HEPG2 cells respectively. On exposure to 3-MC both CYP1A1 and CYP1B1 mRNA levels increased in all three cell lines. However the level of CYP1A1 mRNA in the MOG-G-CCM and CYP1B1 mRNA in the HEPG2 cell line exposed to 3-MC was below the threshold level for basal expression of the transcripts observed in the other two cell lines. Following exposure to 3-MC; MCF7 cells show inducible expression of both CYP1A1 and CYP1B1 protein, HEPG2 cells express CYP1A1 protein and MOG-G-CCM cells CYP1B1 protein. The results of this study suggest that a threshold of expression for CYP1A1 and CYP1B1 mRNA expression must be obtained before expression of protein occurs. This may indicate why although CYP1B1 mRNA is present in normal tissue the protein is not detectable.

References

1. Murray GI, Melvin WT, Greenlee WF, Burke MD. 2001 Annu Rev Pharmacol Toxicol 41: 297.

2. Rochat B, Morsman JM, Murray GI, Figg WD, McLeod HL. 2001 J Pharmacol Exp Ther 296: 537.

Acknowledgement: Research was funded by University of Aberdeen Development Trust.

**P166**

**CONJUGATED LINOLEIC ACID ENRICHED MILKFATS INHIBIT GROWTH AND MODULATE PROSTAGLANDIN PRODUCTION IN THE MCF-7 HUMAN CANCER CELL LINE.**

A. Miller*, C. Stanton[1], J. Murphy[2] and R. Devery.
School of Biotechnology, National Institute for Cellular Biotechnology, Dublin City University, Dublin 9, Ireland; [1]Teagasc Dairy Products Research Centre, Moorepark, Fermoy, Co. Cork, Ireland; [2] Teagasc, Dairy Production Department, Moorepark, Fermoy, Co. Cork, Ireland.

Because of the potential health benefits attributed to CLA, strategies for CLA enrichment of milkfat have been identified with a view to production of CLA-enriched functional foods. Milk enriched in CLA was obtained from cows on pasture supplemented with full fat rapeseeds (FFR) and full fat soyabeans (FFS). Control milkfat was obtained from cows on pasture only. In this study, we compared the potency of the CLA-enriched milkfats with control milkfat to influence growth, eicosanoid production, lipid peroxidation and glutathione levels in human MCF-7 breast cancer cells. Cell numbers, as measured by trypan blue exclusion, decreased (by 48-66 %) following incubation of MCF-7 cells for 4 days with the three different milkfats (added at 1mg/ml) yielding CLA concentrations between 16.9 and 22.6 μg/ml. When the three milkfats were added at different concentrations, to yield a final CLA concentration of 20 μg/ml, significantly ($p<0.05$) lower cell numbers were obtained (42-44 % of control), confirming the anticarcinogenic potency of milkfat CLA. Vaccenic acid, a milk fatty acid may also influence MCF-7 cell growth. When incubated with MCF-7 cells at the concentrations present in the milkfats (31.4 - 46.4 μg/ml) significant ($p<0.05$) decreases in cell growth were observed . Only the FFR milk fat (containing 22.6 μg/ml CLA) significantly ($p<0.05$) altered prostaglandin synthesis at 24h, increasing $PGF_{2\alpha}$ (by 29 %) and decreasing $PGE_2$ (by 22 %). None of the milkfats

 © 2002 Cancer Research UK

influenced 5-HPETE production. Lipid peroxidation, as measured by 8-epi-$PGF_{2\alpha}$ increased ($p<0.05$) by 55-92 % following incubation with the three milkfats. All milkfats significantly decreased ($p<0.05$) glutathione levels by 17-38 % in cytosolic extracts. Lipid peroxidation and depletion in glutathione levels are indicative of a signalling pathway initiating apoptosis. The data suggest that apoptosis may be the mode of cell death induced by the CLA-enriched milkfats in the MCF-7 cancer cell line.

**P167**

**A NEW APPROACH TO BREAST CANCER CONTROL. TURNING CELL CYCLE CONTROLLER GENES INTO CANCER DRUGS**

Valerie Wells* and Livio Mallucci
Cell Signalling and Growth Laboratory, Division of Life Sciences, King's College London, 150 Stamford Street, London SE1 9NN.

Breast cancer is a widespread disease. Like many other cancers, cancers of the breast can be refractory to chemotherapy which may instead remain harmful to normal cells, including those of the immune system and progenitor cells. Our strategy is based on the use of a naturally occurring immunomolecule with a putative role in cancer surveillance, based on its ability to exploit genetic differences between normal and cancer cells. The betaGBP cytokine[1] negatively regulates the cell cycle by activating an S phase checkpoint. Downregulation of tyrosine kinase receptor activated signalling by betaGBP results in altered expression of cell cycle controller genes which in cancer cells, but not in normal cells, activate programmed cell death. In particular the E2F-1 transcription factor has emerged as a key player in promoting apoptosis. Our studies are focused on mammary cancer cells[2] which differ in oestrogen receptors status, expression of the EGF family of receptors and sensitivity to chemotherapeutic drugs. For the present investigation we have selected the following breast cancer cell lines: BT20, T47D, MCF7 and MCF7-D40 (doxorubicin resistant). MCF10a, a line of normal luminal breast cells is used as a comparison. We have found that all the cancer cells, including the drug resistant ones, but not the normal breast luminal cells initiate a death programme after a gap period of 2-5 days from betaGBP enforced $S/G_2$ arrest. We have evidenced two stages in the response to treatment with betaGBP: Stage 1, growth arrest; stage 2, after a varying time-lag, apoptosis. We are examining which genes are responsible for stage 1 and stage 2 responses. Genes involved in stage 1 include those controlling the expression of PI3-kinase signalling and Ras-MAP kinase signalling. Genes involved in stage 2 include those involved in cyclin kinase activity and E2F-1 expression, a gene highly expressed in cancer cells, whose deregulation leads to E2F-1 induced apoptosis. We are manipulating the expression of these genes to identify critical steps in the chain of events activated by betaGBP which may be selected as direct targets in drug development.

1 Blaser, C., Kaufmann, M., Muller, C., Zimmerman, C., Nath, N., Mallucci, L. and Pircher, H. (1998). beta-Galactoside-binding protein secreted by activated T cells inhibits antigen-induced proliferation of T cells. European Journal of Immunology *28*, 2311.

2 Wells, V., Davies, D., and Mallucci, L. (1999). Cell cycle arrest and induction of apoptosis by beta-galactoside binding protein in human mammary cancer cells. A potential new approach to cancer control. European Journal of Cancer *35*, 978.

This work is supported by the Breast Cancer Campaign.

**P168**

**TIP60 CORRELATES WITH THE DIFFERENTIATED PHENOTYPE IN LNCaP PROSTATE CANCER CELLS**

K Halkidou*, S Cook, ME Brady, DE Neal, CN Robson;
Prostate Research Group, Dept. of Surgery, University of Newcastle Medical School, Newcastle-Upon-Tyne, NE2 4HH

Prostate cancer (CaP) is the second most prevalent malignancy in UK men, accounting for more than 10,000 deaths each year. Prostatic tumours are initially androgen-sensitive and regress after androgen ablation. However, most disseminated and highly de-differentiated prostate adenocarcinomas eventually recur, at which point they are termed "androgen refractory", and can no longer be cured by current conventional therapy. We have previously identified Tip60 as a coactivator of the human androgen receptor (AR). We have also shown that nuclear localization of Tip60 inversely correlates with metastasis at the time of diagnosis. The direct link between loss of the differentiated phenotype and the degree of malignancy of the tumour, highlights the importance of studying factors associated with differentiation. Since cellular differentiation is largely a matter of transcriptional regulation, we were intrigued to examine Tip60's involvement in the process of prostate epithelium differentiation.

**MATERIALS AND METHODS**: Western blotting was performed to examine the protein levels of several cell cycle regulatory molecules and differentiation markers in prostate cancer LNCaP cells treated with Trichostatin A (TSA), a histone deacetylase (HDAC) inhibitor. Immunofluorescence staining was also undertaken for those samples. Flow cytometry analysis of Tip60 expression levels and cell cycle profile analysis were performed. Growth assays were also used to demonstrate TSA's effect on cell proliferation. **RESULTS**: TSA causes growth arrest and differentiation in LNCaP cells, an androgen responsive CaP cell line. We observed a downregulation of cyclin D1 and an upregulation of p21 in response to TSA treatment. Cell cycle analysis showed TSA arrested LNCaP cells both at G2/M and G1. Immunofluorescence staining of cytokeratin 18 demonstrated a reorganization of the cytoskeleton upon TSA treatment, together with upregulation and predominantly nuclear expression of Tip60, in a distinct speckled pattern. At the same time, histone 4 hyperacetylation directly correlated with the differentiated phenotype. Co-expression of Tip60 with low molecular weight cytokeratins in primary cultures of normal epithelial prostate cells also indicated a connection between Tip60 and the secretory phenotype in benign prostate cells.**CONCLUSIONS**: Our results indicate that Tip60 may be involved in the differentiation process, as it is upregulated and localizes in the nucleus in LNCaP cells caused to differentiate by TSA treatment. Our hypothesis is that Tip60, a histone acetyltransferase enzyme and AR co-activator, plays an important role in the differentiation course in prostate epithelium, possibly by enhancing AR transactivation in response to differentiating stimuli. This role may be altered as the disease progresses towards the androgen independent status.

**P169**

**BRCA1 MEDIATED CELL APOPTOSIS IN RESPONSE TO TAXOL**

Quinn J.E *, Gilmore PG, McCabe N, Mullan PB, Andrews H, Johnston PG, Harkin DP and Kennedy RD. Oncology Department, Queen's University Cancer Research Centre, Belfast City Hospital, Lisburn Road, Belfast, N.Ireland, BT9 7AB.

BRCA1 is a tumour suppressor gene implicated in the predisposition to early onset breast and ovarian cancer. In our previous work (Mullan and Quinn *et al*., Oncogene, 2001, 20, 6123-6131), we generated cell lines with inducible expression of BRCA1 to evaluate its role in mediating the cellular response to various chemotherapeutic drugs commonly used in the treatment of breast and ovarian cancer. We observed a dramatic interaction between BRCA1 induction and exposure to the antimicrotubule agent, Taxol that functions by inhibiting the depolymerization of tubulin. Induction of BRCA1 in the presence of Taxol resulted in a complete loss of cell viability, an effect that was preceded by a transient arrest at the G2/M phase of the cell cycle and which correlated with BRCA1 mediated induction of GADD45. A proportion of the arrested cells were blocked in mitosis suggesting the involvement of BRCA1 in the activation of both a G2 and a mitotic spindle checkpoint.

Our current focus is to investigate the mechanism of BRCA1 mediated cell death in response to Taxol. We have demonstrated by parp and caspase cleavage assays that this is an apoptotic mechanism and have preliminary evidence to suggest that the mechanism may involve either the JNK kinase and/or p38 apoptotic pathways. We aim to provide further evidence that BRCA1 can mediate the cytotoxic effects of antimicrotubule agents through activation of the JNK or p38/MAPK pathways. In order to do this we have generated cells that express both inducible BRCA1 and dominant negative MEKK3, the common upstream regulator of both JNK and p38. We aim to use these cells to determine if abrogation of these pathways can prevent BRCA1 mediated cell death in response to Taxol. Finally, our data suggests that BRCA1 expression levels in tumours may be an important determinant of response to these compounds.

REFERENCE

BRCA1 and GADD45 mediated G2/M cell cycle arrest in response to antimicrotubule agents. Mullan PB, Quinn JE, Gilmore PM, Mc Williams S,

Andrews H, Gervin C, McCabe N, Song YH, Maheswaran S, Liu E, Haber DA, Johnston PG, Harkin DP. Oncogene (2001) 20, 6123-6131.

**P170**

**ACTIVATION OF THE PROTEASOME AND APOPTOTIC PATHWAYS BY A CACHECTIC FACTOR AND ATTENUATION BY ICOSAPENTAENOIC ACID**

H J Smith* and M J Tisdale, Pharmaceutical Sciences Research Institute, Aston University, Aston Triangle, Birmingham, B4 7ET.

A proteolysis inducing factor (PIF) was isolated from a cachexia - inducing murine tumour (MAC16). The effect on protein degradation through the ubiquitin-proteasome proteolytic pathway as well as the induction of apoptosis in skeletal muscle was determined using C2C12 myotubes as a surrogate model. Proteasome activity was assessed using chymotrypsin activity, and was increased by PIF at 4.2nM ($p<0.005$) after 24h incubation. The expression of alpha and beta subunits of the proteasome 20S core, along with the conjugating enzyme E2, were shown to be increased by Western Blotting of myotube supernatants at a concentration of PIF between 1 and 20nM. This increase in proteasome activity and expression was attenuated by 50μM EPA.

The activity of cysteinyl - directed aspartate specific proteases was also measured fluorometrically in C2C12 myotubes. PIF was shown to stimulate the apoptotic initiating caspases 8 and 9 at 2.1 and 4.2nM ($p<0.01$), and the apoptotic effectors caspases 2, 3 and 6 also at 2.1 and 4.2nM ($p<0.001$). The activation of these caspases was inhibited by 50μM EPA. Western Blotting analysis showed that PIF induced an increased expression of caspase 3, the apoptotic protein Bax and cytochrome c between 1 and 20nM in C2C12 myotubes, an effect that was again attenuated by 50μM EPA. A non-isotopic assay for the quantitation of free nucleasomes in apoptotic cells showed that PIF significantly increased the formation of apoptotic nucleasomes between 1 and 20nM ($p<0.05$) in C2C12 myotubes.

These results suggest PIF increases proteasome activity and induces apoptotic pathways in skeletal muscle, a mechanism that can be attenuated by EPA.

**P171**

**A TUMOUR-DERIVED LIPID MOBILISING FACTOR AFFECTS CHANGES IN CARBOHYDRATE METABOLISM AND FATTY ACID OXIDATION IN CANCER CACHEXIA**

S.T.Russell*[1] and M.J.Tisdale[1]. [1] Pharmaceutical Sciences Research Institute, Aston University, Aston Triangle, Birmingham, B4 7ET.

Changes in carbohydrate metabolism are often observed in animals and patients with cancer. This may be due to demand from the tumour for a fuel source, since solid tumours are anaerobic as a result of poor vascularisation. Tumours have previously been shown to utilise glucose with the production of lactate with a corresponding up-regulation of the Cori Cycle; which is energy depleting and would contribute to the weight loss observed in cancer cachexia.

Previously a tumour-derived lipid mobilising factor (LMF) was isolated from the urine of cachectic cancer patients and was shown to produce weight loss in non-tumour bearing mice. LMF produced direct lipolysis in isolated murine adipocytes suggesting weight loss was specific to loss of adipose tissue. LMF was shown to stimulate glucose uptake in $C_2C_{12}$ myoblasts and also produced a decrease in serum levels of glucose *in vivo*. The effect of LMF on glucose was examined using the 2-Deoxyglucose tracer method, and showed elevated utilisation in the brain, heart, brown adipose tissue (BAT) and white adipose tissue (WAT) of non-tumour bearing mice. There were also increases observed in the metabolic rates (Rg) of a number of tissues, including a 3-fold increase in brain tissue, which may contribute to the decrease in glucose serum levels.

Lipid oxidation was also observed to increase in LMF treated mice, as determined by the conversion of $^{14}$C Triolein into $^{14}CO_2$. There was a 67% increase in oxidation over a 24h period in the LMF treated mice. Lipid accumulation was also observed particularly in the liver, WAT and BAT of the LMF treated group.

These results suggest the changes in carbohydrate metabolism and increase in whole body fatty acid oxidation observed in cachectic cancer patients may arise from the production of LMF.

**P172**

**THE INFLUENCE OF NEP/ECE METALLOPROTEINASE EXPRESSION ON STROMAL-EPITHELIAL INTERACTIONS IN PROSTATE CANCER**

L. A. Dawson*[1], N. J. Maitland[2], A. J. Turner[1], B. A. Usmani[1].
[1]School of Biochemistry & Molecular Biology, University of Leeds, Leeds LS2 9JT; [2] YCR Unit, Dept.of Biology, University of York YO10 5YW

Prostate cancer (PC) is the most prevalent form of male cancer with one in five males currently developing invasive PC. Changes in stromal-epithelial interactions in the developing tumour are believed to contribute to PC progression and metastasis. A mitogenic role for small regulatory peptides including endothelin-1 (ET-1) has been implicated in PC. ET-1 is cleaved from its precursor, big ET, through the actions of endothelin-converting-enzyme (ECE). Neutral endopeptidase (NEP), which has homology to ECE, is known to cleave and inactivate mitogenic peptides such as ET in metastatic PC. In previous studies we have shown elevated levels of ECE-1 within the stroma adjacent to tumor epithelia in primary prostate adenocarcinomas. In this study we propose to investigate the expression of NEP and ECE in primary prostatic epithelium, stromal material and in PC cell lines. This will allow elucidation of the role of these peptidases in modulating stromal-epithelial interactions during PC progression. Matrigel invasion chambers will be used to study the effects of normal and malignant stroma on the invasive capacity of tumour epithelial cells. The cell lines utilised in this study include androgen-sensitive LNCaP, normally transformed PNT-1a, PNT2-C2 and P4E6, and androgen-independent PC-3, PPC-1 and Du145 cells. Preliminary data indicates that stromal cells influence invasion, with increased invasion in the presence of stroma. PC-3 cells are highly invasive compared to PNT2-C2 cells. The addition of NEP to PC-3 and Du145 cells dramatically reduced invasion, with invasive capacity returning to untreated levels on addition of NEP inhibitor (phosphoramidon). Further studies are being conducted in the presence or absence of ET, ET antagonists, NEP, ECE and their inhibitors.

**P173**

**NEUTROPHIL-ENDOTHELIAL INTERACTIONS FOLLOWING ENDOTHELIAL PRE-CONDITIONING BY THE BREAST CARCINOMA CELL LINE, MDA-MB231**

A.C. Brooks[1*], G.B. Nash[2], C.S. Parkins[1], and G.M. Tozer[1].
[1] Tumour Microcirculation Group, Gray Cancer Institute, Mt Vernon Hospital, Northwood, Middx. [2] Dept. of Physiology, Medical School, University of Birmingham

Neutrophils recruited to sites of inflammation, adhere to endothelial cells and evoke vascular injury by undergoing oxidative burst and releasing cytolytic granule contents. It is clear that neutrophil recruitment into tumours plays a major role in vascular-targeted therapies[1]. Using the endothelial flow cell assay, an *in vitro* model of neutrophil-recruitment under conditions of flow, we have previously investigated the effects of the vascular targeting agent combretastatin A4-P on neutrophil recruitment. However, this model currently uses endothelial cells isolated from normal tissue, and not tumours. Studies using tumour-conditioned endothelial cells *in vitro*, and *in vivo* studies of tumour vasculature[2], indicate that leukocyte/endothelial-interactions are decreased in tumours, compared to normal tissue. This process termed "tumour anergy", is hypothesized as a mechanism by which tumours may evade host immune response. Here we investigate conditioning of normal endothelial cells by the breast carcinoma cell line MDA-MB231, and its effects upon neutrophil recruitment in our model system.

HUVEC were seeded into flow-slides as previously described[3], and placed in series with flow-slides containing MDA-MB231 cells and incubated for either 24 or 48 hours. Following conditioning, HUVEC were incubated with either vehicle alone or TNF-α (100 U/ml) for 4 hours prior to neutrophil perfusion. Neutrophil recruitment in each category of the adhesive process (rolling, firm adhesion, and migration) was recorded.

 © 2002 Cancer Research UK

Conditioning by MDA-MB231 cells for 24 hours did not result in alteration of basal neutrophil recruitment to HUVEC treated with vehicle alone. However, a significant decrease in the number firmly adherent, and migrated but not rolling neutrophils recruited to TNF-α–activated endothelium was observed after 5 minutes of neutrophil perfusion into tumour-conditioned slides. This suggests that tumour conditioning affected some factor involved in neutrophil firm adhesion, whilst elements involved in rolling remain unaffected. In contrast, following 48 hours of tumour-conditioning both basal and TNF-α-activated neutrophil recruitment was significantly increased.
The data presented demonstrate that it is possible to condition normal endothelial cells using tumour cells in a modified flow cell assay, and that this conditioning is able to modulate neutrophil recruitment. Further studies will examine the factors involved in these alterations in neutrophil recruitment.

1. Cecic I, Parkins CS, Korbelik M (2001): *Photochem Photobiol* 74: 712-20.
2. Griffioen AW, Molema G: (2000) *Pharmacol Rev* 52: 237-68
3. Luu NT, Rainger GE, Nash GB (1999): *J Vasc Res* 36: 477-85
Supported by the Association for International Cancer Research and Cancer Research UK

**P174**
**PAX3 FUNCTIONS TO PROMOTE SURVIVAL THROUGH DOWNREGULATION OF PTEN**
H. Li,* C. J. Parker, and P. Kumar, Biological Sciences Department, Manchester Metropolitan University, Chester St., Manchester M1 5GD.

The paired box transcription factor PAX3 is expressed in the early embryo in neural crest and developing muscle. Its expression has been demonstrated in the cell lines and tumour tissues of rhabdomyosarcomas (RMS) and melanomas. It has been reported that downregulation of PAX3 can promote apoptosis in primary cultures of melanomas, and PAX3 can upregulate the anti-apoptotic protein, BCL-XL in normal and malignant myocytes. PTEN is known to modulate PI3 kinase, which is involved in cell survival, migration and proliferation. PTEN mutations occur in several tumour types. We have constructed a pTet-On doxycycline inducible C2C12 myoblast cell line transfected with PAX3 and found that after doxycycline induction of PAX3, PTEN was downregulated. When the C2C12 myoblasts were cultured in differentiation medium (2% horse serum in DMEM), doxycycline induction of PAX3 expression caused the downregulation of p27. When C2C12 myoblasts transfected with PAX3 were grown in suspension culture, we found using an Annexin V apoptosis staining kit, that the apoptotic index was reduced compared with control myoblasts. Cell growth rates were reduced ($p<0.01$) when antisense PAX3 was transfected into an embryonal RMS cell line, JR1 and a melanoma cell line, B16F10. These results suggest that PAX3 may function in survival and proliferation in tumours, partly through regulation of the PTEN-PI3K pathway, and may represent a new target for cancer treatment.

**P175**
**EXPRESSION OF ADAM-11 SPLICE VARIANTS IN BREAST CANCER**
C. O' Shea*[1], C. Duggan[1], Y. Buggy[2], E. McDermott[2], A.D.K Hill[2], N. O' Higgins[2], M.J. Duffy[1]. Depts. of Nuclear Medicine[1] & Surgery[2], St. Vincent's University Hospital, Dublin 4, Ireland.

The ADAMs (A Disintegrin And Metalloprotease) are membrane proteins that contain both protease and adhesion domains and thus are potentially important in cancer invasion and metastasis. The gene encoding ADAM-11, located on chromosome 17q21, has 2 splice variants, i.e., ADAM-11-769 and ADAM-11-524. ADAM-11 was reported to be somatically rearranged in 2 primary breast cancers and was postulated to be a candidate tumour suppressor gene. The aim of this study was to investigate the distribution and potential clinical significance of ADAM-11 (total) and its splice variants in breast cancer. mRNA for total ADAM-11, ADAM-11-769 and ADAM-11-524 were measured using RT-PCR. The splice variants were separated on Spreadex EL 1200 DNA gels. No difference was found in the frequency of expression of ADAM-11 (total) or ADAM-11-769 in normal breast tissue (n=23), fibroadenomas (n=39), primary breast cancers (n=109) and nodal metastases (from breast cancer) (n=8). However, within the ADAM-11 mRNA-positive tissues, ADAM-11-524 tended to be expressed more often in normal breast tissue (5/12, 42%) and fibroadenomas (9/27, 33%) compared to the breast cancers (13/70, 19%) and nodal metastases (0/4, 0%). In addition, the relative levels of ADAM-11-524 (arbritary units/ADAM-11-769) were approximately 4 times higher in normal breast tissue compared to the cancers (p=0.0691). Significantly higher levels of ADAM-11 (total) were detected in grade 1 and 2 cancers compared to grade 3 tumours (p=0.0393). Moreover, expression levels of ADAM-11 (total) in the carcinomas correlated inversely with MMP-1 mRNA levels (n=23, r=-0.485, p=0.0228), MMP-2 (active) protein levels, as determined by zymography, (n=22, r=-0.564, p=0.0097) and uPA protein levels, as determined by ELISA, (n=60, r=-0.349, p=0.0074). An inverse relationship was also found between the frequency of expression of ADAM-11-524 and uPA mRNA (p=0.004). In conclusion, our results show that ADAM-11-524 is expressed less frequently in breast cancer than in non-malignant breast tissue. The clinical significance of this decreased expression remains to be determined.

**P176**
**SURVIVIN mRNA AND PROTEIN IN BREAST CANCER**
B. Ryan*[1], C. O' Shea[1], N. O' Donovan[2], S. Kennedy[3], J. Crown[2], E. McDermott[4], A.D.K Hill[4], N. O' Higgins[4], M.J. Duffy[1]. Depts. of Nuclear Medicine[1], Medical Oncology[2], Pathology[3] & Surgery[4], St. Vincent's University Hospital, Dublin 4, Ireland.

Survivin is a 16.5 kDa protein that both suppresses apoptosis and regulates cell division. Early data suggested that the survivin gene was rarely expressed in normal tissue, but was widely expressed in malignancy. These findings suggested that survivin might be a new candidate marker for breast cancer. The aim of this investigation was to compare the expression of survivin in benign and malignant breast tissue. Survivin expression was measured at the protein level using both ELISA and Western blotting and at the mRNA level using RT-PCR. Using ELISA, higher levels of survivin protein were detected in both primary breast carcinomas (n=70) and nodal metastases (from breast cancer) (n=16) compared with fibroadenomas (n=16) (p=0.0069, p=0.0131, respectively). Using Western blotting, survivin protein was detected in a higher proportion of nodal metastases (19/19, 100%) and carcinomas (69/77, 90%) than fibroadenomas (18/26,69%) (p=0.0226, p=0.0300, respectively). Similarly, survivin mRNA levels were significantly higher in the cancers compared to the fibroadenomas (p=0.0001). In addition, frequency of expression of survivin protein, as measured by Western blotting, tended to be higher in mRNA-positive tumours (24/25, 96%) than mRNA-negative cancers (2/4, 50%) (p=0.0515). Expression levels of survivin protein in the carcinomas, as determined by ELISA, correlated positively with tumor grade (n=61, r=0.432, p=0.0007) and inversely with estrogen receptor (n=63, r=-0.316, p=0.0121) and progesterone receptor (n=64, r=-0.380, p=0.0028) concentrations. Higher levels of survivin protein were also found in ductal carcinomas compared to lobular cancers (p=0.0424) However, no significant relationship was found between survivin protein and either axillary nodal involvement or tumor size. We conclude that while survivin is detected in the majority of carcinomas, it is also present in benign breast tissue. Further work is necessary to establish a possible clinical value for survivin in breast cancer.

**P177**
**INVOLVEMENT OF TIP60 IN ANDROGEN RECEPTOR SIGNALLING**
Vasileia Sapountzi*, Ian R. Logan, David E. Neal, Craig N. Robson
Prostate Research Group, School of Surgical Sciences, Medical School, University of Newcastle Upon Tyne, Newcastle Upon Tyne, NE2 4HH, United Kingdom

Prostate cancer is one of the most common causes of death for males in Western countries. In the initial stages of the disease, carcinoma is essentially curable and exhibits androgen dependent growth. In the later stages of the disease however, it can develop androgen independence that often results in death. Androgen receptor mediated signalling pathways are critical in normal prostate development and play an important role in the progression of

© 2002 Cancer Research UK

prostate cancer. Androgen receptor (AR) is a transcription factor activated by androgens but also by hormone independent phosphorylation through various kinase pathways. AR mediates its effect on gene expression with the aid of coregulatory molecules. One AR coactivator is TIP60, a protein initially found to interact with the Tat protein of HIV-1(1, 2). Several coactivators are at least partially regulated by phosphorylation.

In this study, growth factor signalling pathways including the fibroblast growth factor (FGF) and the epidermal growth factor (EGF), and pathways involving protein kinase A (PKA), protein kinase C (PKC), mitogen activated protein kinases (MAPK) and $Ca^{2+}$- dependent kinases (CaMK) were screened to see if they affect TIP60 transcriptional activity. We demonstrate that PKA affects TIP60 transcriptional activity, as shown by mammalian one-hybrid assays and this effect is diminished by dominant negative PKA suggesting that the effect of PKA on TIP60 is dependent on the catalytic activity of PKA. This is of particular interest as PKA has been shown to activate AR through phosphorylation (3). Finally, EGF, but not FGF1 or FGF2, appears to alter TIP60 activity. These results, in combination with previous findings that protein kinase pathways activate AR, highlight the importance of TIP60 in AR mediated transactivation and implicate phosphorylation as an important mechanism in androgen independent activation of AR signalling.

1.Kamine, J., Elangovan, B., Subramanian, T., Coleman, D. & Chinnadurai, G. (1996) *Virology* 216, 357-66.
2.Brady, M. E., Ozanne, D. M., Gaughan, L., Waite, I., Cook, S., Neal, D. E. & Robson, C. N. (1999) *J Biol Chem* 274, 17599-604.
3.Nazareth, L. V. & Weigel, N. L. (1996) *J Biol Chem* 271, 19900-7.

### P178

### EFFICACY & STABILITY OF A PSA ENHANCER-PROMOTER CONSTRUCT IN PROSTATE CELL LINES & PRIMARY PROSTATE TISSUE

James G. Young[1], James Latham[1,] Peter F. Searle[1], Alan Doherty[2], D. Michael A. Wallace[2], Lawrence S. Young[1], Nicholas D. James[1]*.
(1). CRC Institute for Cancer Studies, Vincent Drive, Edgbaston, Birmingham B15 2TA. (2). University Hospital Birmingham.

**Introduction and Objectives:** The genes for the prodrug converting enzyme *E. Coli* Nitroreductase (NR) and the reporter gene enhanced Green Fluorescent Protein (eGFP) have been cloned into adenoviral gene therapy vectors. The transgenes are controlled by a double PSA enhancer & single PSA promoter transcriptional regulatory region (TRR) termed "PSAeep". The activity of this promoter was investigated in prostate cancer cell lines as well as primary prostate tissue. The stability of this PSA TRR was examined by Southern blotting. **Methods:** The human cell lines LNCaP, PC-3 and DU145, and the mouse prostate cancer cell lines TRAMP C1 and C2 were infected with adenovirus containing the reporter transgene eGFP and expression assessed by UV microscopy and FACS analysis. Thin sections of cultured primary prostate tissue from radical prostatectomy specimens were infected with adenovirus, and transgene expression analysed. Apoptosis induced by the combination of nitroreductase expressing adenovirus and CB1954 was visualised with the cytokeratin 18 antibody M30. Southern blotting of restriction enzyme digests of adenoviral DNA was performed to assess stability of the TRR. **Results:** GFP expression was detected in all human but not the TRAMP cell lines on UV microscopy and FACS analysis. Gene expression levels in the cell lines PC-3 & DU145 were lower than in LNCaP from the PSAeep TRR. Primary prostate tissue infections confirmed eGFP expression exclusively from glandular epithelium. Apoptosis was also confined to glandular epithelium with NR expressing adenovirus and CB1954. Southern blotting of viral DNA digests showed instability of this PSA TRR, with evidence of recombination to form single and triple enhancer variants during viral replication. **Conclusions:** Epithelial specificity of the PSA promoter-enhancer construct was confirmed in primary prostate tissue. Genetic instability with evidence of homologous recombination was found in viral DNA, making this vector unsuitable for clinical trials. Novel methods to increase the strength of PSA TRR driven gene therapy will have to be found as replication of promoter or enhancer elements leads to genetic instability in vectors.

### P179

### DISULFIRAM INHIBITS 5-FU-INDUCED NF-κB ACTIVITY AND ENHANCES CYTOTOXICITY OF 5-FU TO HUMAN COLORECTAL CANCER CELL LINES

Weiguang Wang[1*], Howard L. McLeod[2] and James Cassidy[3]
[1]Department of Medicine and Therapeutics, University of Aberdeen, Aberdeen, AB25 2ZD, UK [2]Washington University School of Medicine, St. Louis, MO 63110-1093 USA and [3]Beatson Laboratories, Glasgow G61 1BD, UK

Colorectal cancer (CRC) is the third leading cause of cancer deaths in developed nations. 5-fluorouracil (5-FU) is the first line agent for CRC chemotherapy. Resistance of cancer cells to 5-FU induced apoptosis is considered the major mechanism of chemoresistance leading to the failure of CRC chemotherapy. NF-κB is an anti-apoptotic transcription factor. Cancer cells with high NF-κB nuclear activity demonstrate robust chemo- and radioresistance. Disulfiram (DS) is an anti-alcoholism drug inhibiting TNFα induced NF-κB activity in T lymphocytes. The aim of this study is to investigate the influence of DS on NF-κB activity in CRC cell lines and effect of DS on the cytotoxicity of 5-FU to these cell lines. The nuclear NF-κB activity in CRC cell lines DLD-1 and RKOwt was significantly induced by 5-FU treatment in a concentration- and time-dependent manner. NF-κB subunits p50 and p65 were involved in 5-FU-induced NF-κB activity. Compared to drug-sensitive cells, Thymidylate synthase inhibitors (5-FU and Tomudex) resistant cell lines ($H630_{5FU}$, $H630_{TDX}$, $RKO_{TDX}$, $W1L2_{TDX}$ and $R10_{5FU}$) demonstrated higher constitutive NF-κB nuclear activity. The protein and mRNA of NF-κB p50 and p65 subunits were overexpressed in these cell lines but no DNA amplification was detected. 5-FU induced IκBα degradation, promoted NF-κB nuclear translocation but not its DNA binding affinity. 5-FU treatment did not influence the activities of AP-1, AP-2, Oct-1, SP-1, CRE-B and TFIID. DS strongly inhibited constitutive and 5-FU-induced NF-κB activity in a dose-dependent manner. DS inhibited both NF-κB nuclear translocation and DNA binding affinity but has no effect on 5-FU-induced IκBα degradation. Used in combination, DS significantly enhanced the apoptotic effect of 5-FU on DLD-1 and RKOwt cell lines and synergistically potentiated the cytotoxicity of 5-FU to both cell lines. As well, DS can reverse the resistance of $H630_{5FU}$ cells to 5-FU. As an authorised anti-alcoholism drug, the pre-clinical and clinical data of DS are available. It is relatively simple to translate its anticancer usage from *in vitro* experiment to clinical trial.

### P180

### SERIAL MOLECULAR ANALYSIS OF BCR-ABL EXPRESSION AND CHIMERISM STATUS IN CML PATIENTS FOLLOWING STI571 TREATMENT.

Gately K.*, Ryan J., McCann S., Howard J., Stallings R., Carroll P., Browne P, Conneally E, Lawler M. Department of Haematology St James's Hospital and Trinity College Dublin; National Centre for Medical Genetics, Our Lady's Hospital for Sick Children, Crumlin, Dublin, Ireland

Chronic Myelogenous Leukemia (CML) is a stem cell malignancy which is characterised by the disease specific marker, the Philadelphia chromosome. This is a result of the translocation t(9;22) which generates the *bcr-abl* fusion gene encoding a leukemia specific tyrosine kinase p210. Enhanced tyrosine kinase activity by p210 contributes to the abnormal intracellular signalling, defective adhesion and resistance to apoptosis characteristic of CML cells. STI 571 is a tyrosine kinase inhibitor which has been proven effective in the treatment of CML. Serial molecular analysis is being performed in 35 patients receiving STI571(Novartis) at St James's Hospital. Peripheral blood and bone marrow are collected from patients prior to STI treatment and at regular intervals during treatment. Lineage selection is performed on all samples to isolate populations of $CD34^+$, $CD33^+$ and $CD15^+$ cells using magnetic bead selection. Molecular studies have been designed to measure changes in *bcr-abl* expression (as a marker of STI571's efficiency as an anti leukemia drug). A quantitative real time RT-PCR assay has been developed to detect *bcr-abl* and has a sensitivity of detection of 1 leukaemia cell in a background of $10^6$ normal cells. Real time RT-PCR analysis indicates substantial downregulation of bcr-abl mRNA at 6 months in patients with haematological and cytogenetic responses (n = 17 ). Four patients in the study had relapsed following allogeneic stem cell transplantation (SCT). Two

© 2002 Cancer Research UK

patients received STI 571 directly following relapse while in a further 2 patients, a partial response was seen following immunotherapy using donor lymphocyte infusions (DLI) from the original donor. Both of these patients relapsed and subsequently received a non myeloablative stem cell transplant. In both patients a partial response was seen but the patients subsequently relapsed and received STI 571. All 4 patients are currently being monitored both by quantitative RT-PCR of *bcr-abl* and chimerism analysis to ascertain the origin of hemopoesis (donor or recipient) using short tandem repeat PCR of monoclonal antibody selected haemopoetic lineages. In the 2 patients who received STI 571 following relapse after allogeneic SCT, serial analyses of chimerism and *bcr-abl* positivity have shown reversion to donor chimerism in all lineages and *bcr-abl* negativity following treatment with STI. The 3rd patient showed a partial response with reduction in *bcr-abl* transcripts and 70% donor chimerism while in the 4th patient there was no significant reduction in *bcr-abl* transcripts and chimerism analysis indicated 20-30% donor cells in all lineages tested. Thus STI treatment leads to a significant reduction in *bcr-abl* transcripts in CML patients in chronic phase although no patient has achieved complete *bcr-abl* negativity. In patients receiving STI-571 following relapse after allogeneic SCT, molecular analysis using both real time PCR and chimerism analysis indicates that complete clearance of bcr-abl transcripts and reversion to donor hemopoiesis can be achieved.

### P181
### GLYCINE-EXTENDED GASTRIN STIMULATION OF MMP-2 SECRETION IN DIFFERENT CELL TYPES IS NOT SPECIFIC TO ONE ISOFORM OF THE CCK-2 RECEPTOR

RA Dean*, PA Clarke, SA Watson
Academic Unit of Cancer Studies, QMC, University Hospital, Nottingham, NG7 2UH

**Background & Aims:** Gastrin has growth promoting effects on both normal and malignant tissues of the gastrointestinal tract. The cholecystokinin/gastrin receptor (CCK-2R) can occur in several isoforms, which may have roles to play in a gastrin autocrine pathway in malignant cells. Glycine-extended gastrin (Gly-G17) has been shown to promote matrix metalloproteinase (MMP) secretion and therefore we are investigating whether the CCK-2 isoforms have different effects on MMP secretion.
**Methods:** Gelatin zymography was used to investigate the protein expression of MMP-2 in eight cell lines with or without the stimulation of GlyG17 ($10^{-7}$M). Western blotting was used to characterise which type of CCK-2R isoform each cell line expressed, using antibodies specific for either the classical or truncated CCK-2 receptor. The cell lines investigated were the human fibrosarcoma HT1080 transfected with MT1-MMP, the mouse fibroblast NIH 3T3 line transfected with the classical CCK-2 or truncated CCK-2 receptor, and the human gastro-intestinal tumour cell line AGS transfected with classical CCK-2R, as well as all their respective vector control cell lines.
**Results:** A significant increase in pro and active MMP-2 ($p<0.05$) and proMMP-9 ($p<0.05$) at the protein level, following stimulation by GlyG17 was observed in the human fibrosarcoma cell line transfected with MT1-MMP. This transfected cell line had a 2.7 fold increase in the expression of a ~98kDa band when blotted for classical CCK-2R, compared to its vector control. The truncated CCK-2 receptor NIH 3T3 transfectants had a significant increase ($p<0.05$) in proMMP-2 and expressed a ~68kDa band when blotted for the truncated CCK-2R. Neither of the two classical CCK-2R transfectants, (AGS and 3T3) showed a significant change in MMP-2 secretion.
**Conclusion:** GlyG17 has previously been shown to increase protein levels of pro and active MMP-2 in a human colonic cancer cell line. We have shown that this stimulation by GlyG17 is mediated through either classical or truncated CCK-2R although presence of receptor does not confirm the effect. Further research needs to be undertaken to determine the mechanism by which Gly-G17 can increase MMP-2 secretion and or activation.

### P182
### TRANSFECTED MOSAIC SPHEROIDS: A NEW MODEL FOR EVALUATION OF TUMOUR CELL KILL IN A TARGETED RADIOTHERAPY/GENE THERAPY STRATEGY UTILISING RADIOASTATINE.

*M Boyd[1], SC Ross[1], P Welsh[2], G Akubani[2], MJ Zalutsky[3] and RJ Mairs[1].
1. Department of Radiation Oncology, Cancer Research UK Beatson Labs, Glasgow, G61 1BD
2. Department of Radiology, Duke University Medical Centre, North Carolina, USA, 27710.

Aims
Targeted radiotherapy is the selective irradiation of tumour cells by radionuclides conjugated to tumour-seeking molecules. The most promising non-immunological agent is radiolabelled MIBG which is actively taken up via the noradrenaline transporter (NAT) in a narrow range of NAT expressing tumours. To apply MIBG targeting for a wider range of tumours, we introduced NAT cDNA into non-NAT expressing UVW glioma cells and achieved high levels of [$1^{31}$I]MIBG uptake and dose-dependent cell kill. Cell kill has been demonstrated in three-dimensional tumour spheroids, which succumb to [$^{131}$I] MIBG mediated cell kill more readily than monolayers due to the radiation cross-fire, which is an added benefit of this strategy. To assess the magnitude of radiation mediated bystander effects, we have developed an *in vitro* tumour model, transfected mosaic spheroids (TMS), which allow modelling of gene therapy strategies when less than 100% of the spheroid express the therapeutic transgene.
Methods
TMS are spheroids composed of two cell populations derived from a single parent cell line. One population of cells are transfected with NAT, the other with GFP as a marker for non-transfection. Spheroids composed of various proportions of NAT expressing cells were prepared by mixing the correct proportions of the two cell populations in a spheroid spinner flask. TMS were then dosed with of [$^{131}$I]MIBG and [$^{211}$At]MABG and cytotoxicity determined by clonogenic assay and spheroid regrowth delay.
Results
Cell killing in TMS by [$^{131}$I]MIBG and [$^{211}$At]MABG, increased in direct proportion to the fraction of NAT-transfected cells and dose administered. When only 5% 0f the cells within the spheroid expressed NAT, the spheroids were sterilised after administration of 14MBq/ml [$^{131}$I]MIBG. [$^{211}$At]MABG was 700X more radiotoxic with spheroid sterilisation occurring after administration of only 20kBq/ml [$^{211}$At]MABG.
Conclusions
No current gene delivery tool allows introduction of transgenes to every cell within a tumour. Cancer gene therapy strategies must therefore have a component of collateral cell kill. We developed a TMS model to allow quantification of bystander effect and utilised it to demonstrate the efficacy of a novel targeted radiotherapy/gene therapy strategy utilising astatinated MABG, which allows spheroid sterilisation when only 5% of the tumour mass expresses NAT. This level of gene transfection should be readily attainable *in vivo* and suggests that this system may be effective for the treatment of cancer.

### P183
### VINORELBINE INDUCES p38 BUT NOT MAPK ACTIVATION NOR p53 EXPRESSION IN BREAST CANCER CELL LINES

AA Liem*[1], MVCL Appleyard[1], MA O'Neill[1], TR Hupp[2], MP Chamberlain[3], CR Wolf[3] and AM Thompson[1], [1]Dept. of surgery & Molecular Oncology,[2]Dept. of Molecular & Cellular Pathology,[3]Biomedical Research Centre, University of Dundee, DD1 9SY,U.K.

**Background** To overcome the problem of drug resistance in breast cancer treatment, combination chemotherapy is administered. The combination of vinorelbine and doxorubicin in the treatment of metastatic breast cancer patients has shown higher overall response rates and even vinorelbine as a single agent in patients previously exposed to anthracyclines has resulted in a significant remissions. This could be due to mechanisms involved in the multidrug resistance phenomenon. Alterations in signal transduction and mutant p53 are thought to play a role in drug resistance. Cross-talk has been suggested between these signal transduction pathways and p53. To establish the effect of doxorubicin and vinorelbine upon signal transduction and p53,

© 2002 Cancer Research UK

as single agents, in combination, and as pretreatments, an *in vitro* study was performed using MDA-MB-468 and MCF-7 breast cancer cell lines.
**Materials and Methods** Using the $IC_{50}$ value for vinorelbine and doxorubicin, MDA-MB-468 and MCF-7 were treated for a total of 4 hours. While maintaining the Vinorelbine treatment for 3 hours, doxorubicin was added either 1 hour before (pretreatment doxorubicin), 1 hour after (pretreatment vinorelbine), or at the same time (combined). Single agent controls were set up during pre- and combined treatment. p38 and MAPK activity was determined by kinase assay for the appropriate substrate. p53 expression was determined by western blotting in MCF-7 cells following treatment for 4 and 24 hrs with both drugs using the $IC_{30}$ values as single agent or in combination.
**Results** A higher activity of p38 following vinorelbine-based, but not doxorubicin-based, treatment was demonstrated in both cell lines. This higher activation was noted irrespective of whether vinorelbine was given as a pretreatment or simultaneously with doxorubicin. MAPK activity and p53 expression remained unchanged following vinorelbine treatment.
**Conclusion** Higher p38 activity has been linked previously to p53 phosphorylation, resulting in apoptosis or cell cycle arrest. Our findings, however, suggest a model where p38 activity and p53 expression function independently in response to chemotherapy treatment in breast cancer.

**P184**
**IDENTIFICATION OF DIFFERENTIALLY EXPRESSED mRNAS ASSOCIATED WITH CHROMOSOME 11 TRANSFER INTO THE OVARIAN CANCER CELL LINE OVCAR 3**
*Blenkiron C.[1], Stronach E.A.[1], Sellar G.C.[1] Rabiasz G.J.[1], Miller E.P.[1], Porteous D.J.[2], Smyth J.F.[1], Gabra H.[1] [1]Cancer Research UK Medical Oncology Unit, [2]Medical Genetics section, Department of Medical Sciences, University of Edinburgh, Molecular Medicine centre, Western General Hospital, Edinburgh, EH4 2XU.

Microcell-mediated transfer of chromosome 11 to a clonal derivative of ovarian cancer cell line OVCAR3 produced microcell hybrids which display suppression of growth and cellular migration *in vitro* and inhibition of tumour growth *in vivo*.
Subsequently, mRNA populations from OHN, a clonal derivative of the OVCAR3 parent line, and from 11OH2.1, a growth suppressed microcell hybrid, were used for expression difference analysis by Differential Display RT-PCR (DDRT-PCR), cDNA-Representational Difference Analysis (cDNA-RDA) and cDNA high density filter array (HDFA). These techniques identified genes both up and down regulated with respect to growth.
Quantitative real time RT-PCR was used to validate all expression differences in X transcripts. We identified, in total, 12 validated products upregulated in suppressed clone 11OH2.1 and 4 validated products that were downregulated.
The genes downregulated in association with growth suppression, and therefore of potentially oncogenic function, were RALDH2, IGFBP2 and 2 novel cDNAs. When examined on cell line and primary tumour panels, these genes did not however appear to demonstrate a global increase in expression over normal ovarian expression.
Of the 12 genes upregulated in association with growth suppression, 4 were localised on chromosome 11. These were cathepsin D, proteasome subunit PSMD13, ribosomal subunit RPL27a and αB crystallin. All were shown to have decreased expression in several of the cell lines and primary tumours tested. Furthermore, a tight correlation was observed between the expression of PSMD13 and RPL27a in cell lines and primary ovarian tumours. The significance of this association is being investigated. It is clear that the coupling of functional and expression difference analysis approaches can identify contextually important gene expression underlying cancer phenotypes.

**P185**
**MOLECULAR MECHANISMS OF CD40-INDUCED CELL SURVIVAL IN PRIMARY MALIGNANT B-CELLS**
Claire Dallman*, Peter WM Johnson and Graham Packham,
CRC Wessex Medical Oncology Unit, Cancer Sciences Divison, University of Southampton, Southampton General Hospital, Southampton, SO16 6YD, UK.

**Introduction:** CD40 is a potent survival signalling molecule for normal B-cells and some B-cell malignancies. Activation of CD40 promotes proliferation, B-cell activation and rescue from apoptosis. However, in some cellular settings CD40 ligation induces growth arrest and apoptosis. CD40 stimulation regulates the expression of apoptosis controlling molecules, including A20, Survivin and the Bcl-2 family members, Bcl-$X_L$, Mcl-1 and Bfl-1. However, few studies have analysed the expression of these CD40 targets in primary lymphoid cells.
**Methods:** Following ethical approval (Southampton & S. West Hants LREC Submission 158/00), malignant B-cells were isolated from lymph nodes, spleens or blood samples using density centrifugation and immunomagnetic depletion of non B-cells. We studied low and intermediate grade lymphomas and chronic lymphocytic leukaemias. B-cell purity was determined by flow cytometry. The response of isolated B-cells to CD40 stimulation was determined by treatment with soluble recombinant CD40 ligand (CD40L). Cell viability was determined by propidium iodide exclusion. Isolated B-cells were cultured with and without CD40L for up to 72 hrs. Expression of apoptosis regulators was analysed by immunoblotting and semi-quantitative RT-PCR. There are multiple Bcl-X promoters and their relative activity was measured using specific primers.
**Results:** CD40L suppressed apoptosis in all B-cell malignancies tested, accompanied by significant increases in Bcl-$X_L$ protein expression. Bcl-X promoters were differentially regulated by CD40. Mcl-1 protein expression was also increased or maintained by CD40 stimulation, independent of changes in Mcl-1 RNA levels. Although Survivin, A20, Bfl-1 and Bnip3 were regulated by CD40 in some samples, this was not consistent.
**Conclusions :** CD40 ligation promotes cell survival in all primary B-cell malignancies studied to date. The anti-apoptotic proteins , Bcl-$X_L$ and Mcl-1 are consistently regulated by CD40 whereas other molecules, suggested to be important from studies of cell lines, are only regulated in a subset of samples. Further work will involve analysing more samples and normal B-cell controls and determining the contribution of Bcl-$X_L$ and Mcl-1 using antisense approaches. The mechanisms by which CD40 regulates the Bcl-X promoters will be an area of further research and may help to identify targets for the treatment of B-cell malignancies.

**P186**
**CASPASE DEPENDENT CLEAVAGE OF THE ANTI-APOPTOTIC MCL-1 PROTEIN**
J. Michels*, J. W. O'Neill+, R. Craig*, P. W. M. Johnson, G. Packham
CRC Wessex Medical Oncology Unit, University of Southampton, Southampton, UK, *Dartmouth Medical School, Hanover, USA, + Division of Clinical Research, Fred Hutchinson Cancer Research Center, Seattle, USA

**Introduction:** Mcl-1 is a member of the Bcl-2 family with predominantly anti-apoptotic functions. It is down regulated in many cell systems early during apoptosis which may be important for effective cell killing. This down regulation is often in contrast to other anti-apoptotic family members like Bcl-2 and Bcl-XL. Overexpression of Mcl-1 can increase the apoptotic threshold to various cytotoxic insults. Transgenic mice overexpressing Mcl-1 develop lymphoma with a high frequency. Here we have investigated mechanisms that control the expression of Mcl-1 protein during apoptosis.
**Methods:** Cell lines were treated with cytotoxic agents in the presence or absence of the caspase inhibitor ZVAD-fmk. Mcl-1 expression was determined by immunoblotting and induction of apoptosis was confirmed by analysis of PARP cleavage, a caspase target. To determine susceptibility to in vitro caspase cleavage human Mcl-1, Bcl-2 and Bcl-XL proteins were generated by in vitro translation and incubated with recombinant caspase 3. Mcl-1 cleavage sites were determined by sequencing of caspase 3-derived cleavage products and these sites were mutated by introduction of an Asp>Ala substitution. The susceptibility of wild type and mutant Mcl-1 proteins to in vitro caspase 3 mediated cleavage was determined.
**Results:** Mcl-1 expression was down regulated during apoptosis in several cell systems, concomitant with PARP cleavage. Down regulation is caspase dependent since it was prevented by ZVAD-fmk. Mcl-1 is cleaved efficiently in vitro by caspase 3, whereas Bcl-2 and Bcl-XL are not. Caspase 3 cleavage sites were mapped to Asp127 and Asp157; mutation of these sites

 © 2002 Cancer Research UK

prevented caspase cleavage. These cleavage sites are evolutionary conserved in mouse, rat and zebra fish.

**Conclusions:** Our data confirm that Mcl-1 is down regulated in various cell systems in a caspase dependent manner during apoptosis. We have determined two caspase 3 cleavage sites within Mcl-1 and Mcl-1 is therefore likely to be a direct substrate for caspase cleavage in vivo. We are presently investigating if caspase resistant Mcl-1 mutations can alter the apoptotic response of cells to cytotoxic stimuli and if caspase cleavage of Mcl-1 is the main mechanism of down regulation of Mcl-1 during apoptosis.

**P187**

**BAG-1 PREVENTS STRESS-INDUCED APOPTOSIS AND LONG TERM GROWTH INHIBITION IN BREAST CANCER CELLS VIA AN HSC70/HSP70-DEPENDENT PATHWAY**

Graham Packham*, Ramsey Cutress, Paul Townsend
CRC Wessex Medical Oncology Unit, Cancer Sciences Division, School of Medicine, University of Southampton, Southampton General Hospital, Southampton, SO16 6YD

**Introduction.** BAG-1 is a survival protein that interacts with a wide range of cellular targets. Although there is strong evidence that increased expression of BAG-1 may play an important role in breast cancer, the function of BAG-1 in breast cancer cells has not been studied in detail.

**Procedures.** We overexpressed BAG-1 in MCF7 breast cancer cells to determine its effects on apoptosis and proliferation. We used BAG-1 mutants to identify key amino-acid residues required for BAG-1 function.

**Results.** BAG-1 overexpression completely protected MCF7 cells from apoptosis and long term growth inhibition induced by heat shock. BAG-1 overexpression also partially protected cells from other cell stresses, including hypoxia, radiation and chemotoxic drugs. BAG-1 function required a conserved lysine in the BAG-1 N-terminal ubiquitin-like domain, thought to be important for interaction with the proteasome. BAG-1 function was also dependent on C-terminus residues important for interaction with 70 kDa heat shock protein, HSC70 and HSP70.

**Conclusions.** BAG-1 overexpression confers a broad resistance to stress in breast cancer cells. BAG-1 may function by linking chaperone molecules with the proteasome, to regulate or facilitate protein degradation.

**P188**

**FUNCTIONAL COMPLEMENTATION ANALYSIS OF CHROMOSOME 1 IN NEUROBLASTOMA**

Boulos N[1*], Newbold RF[2], Trott DA[2], Pearson ADJ[3], Tilby MJ[1], Lunec J[1]
[1]Cancer Research Unit, Newcastle University, Newcastle upon Tyne, NE2 4HH, UK
[2]Brunel Institute of Cancer Genetics and Pharmacogenomics, Uxbridge, UB8 3PH, UK
[3]Department of Child Health, RVI, Newcastle Upon Tyne, NE1 4LP,UK

**Introduction:** Neuroblastoma (NB) is the most common extracranial solid tumour of infancy and the third most frequent malignant tumour in children accounting for 15% of all childhood cancer deaths. Chromosome 1p deletions and *MYCN* gene amplification in NB are common genetic abnormalities and correlate with a poor prognosis. 1p deletions are found in approximately 30% to 40% of primary NB cases while *MYCN* amplification is seen in 20% to 30% of cases. Deletion mapping studies and somatic cell fusion experiments suggest that multiple tumour suppressor genes (TSG) located on 1p are involved in NB development and possibly control *MYCN* expression. To date, no specific TSG has been identified to be lost or inactivated in NB, nor has a causal link between 1p loss and the malignant phenotype been established.

**Methods:** Functional complementation analysis for the presence of a TSG(s) that plays a role in NB was conducted using microcell mediated chromosome transfer (MMCT). Microcells were generated from mouse hybrid cell lines that carry either a whole human chromosome 1 tagged with the hygromycin resistance gene or a fragment (1p36-1q23) tagged with the neomycin resistance gene (located at 1p35-1p34). Microcells were fused with IMR-32 neuroblastoma cells that have a 1p deleted and *MYCN* amplified status. Resistant hybrids were selected. A panel of informative chromosome 1 microsatellite markers determined the extent of chromosome 1 incorporated into the hybrids.

**Results:** Fourteen hygromycin resistant IMR-32 hybrids were generated, four of which show marked differentiation *in vitro*. A further hybrid that failed to grow indefinitely exhibited a particularly extreme degree of neurofilament development. All the established hybrids carried material from 1q and proximal 1p regions but none acquired additional material from distal 1p regions. PCR for the hygromycin resistance gene and FISH analysis using chromosome 1 paint and chromosome 1 centromeric marker were performed to further confirm the transfer of chromosome 1. Fusion of microcells carrying the 1p36-1q23 fragment with IMR-32 cells did not generate any viable hybrids.

**Conclusion:** These results are consistent with the hypothesis that normal 1p is not compatible with the genetic background of cells carrying *MYCN* amplification. The map of the transferred chromosome 1 regions indicates selection against the incorporation of distal 1p regions into IMR-32 cells. Moreover, the morphology of the hybrids suggests that one or more regions on chromosome 1 may influence differentiation of NB. More detailed mapping studies will reveal whether a particular region of chromosome 1 is associated with the differentiation.

**P189**

**ANALYSIS OF BRCA1 AND BRCA2 GENES IN THE IRISH POULATION USING DENATURING HIGH PERFORMANCE LIQUID CHROMATOGRAPHY (dHPLC).**

Bronagh O'hIci[1*], Nicola Miller[1,2], Trudi McDevitt[1], Nuala Cody[1], Peter A. Daly[2], Des Carney[3], Enda McDermott[4], Andrew J. Green[1] and David E. Barton[1].
**1.** National Centre for Medical Genetics, Our Lady's Hospital for Sick Children, Crumlin, Dublin 12, Ireland and University College Dublin, Dept. of Medical Genetics. **2.** St. James's Hospital, Dublin. **3.** Mater Misericordiae Hospital, Dublin. **4.** St. Vincent's Hospital, Dublin.

Mutation analysis of disease related human genes requires highly sensitive, specific and reproducible tecniques. Many of the techniques currently in use are time consuming and laborious. The recent development of denaturing high performance liquid chromatography (dHPLC), which is based on heteroduplex detection promises to fulfill these requirements. This technique exploits the differential retention time of homoduplex and heteroduplex fragments on an alkylated non-porous matrix under conditions of partial heat denaturation. This allows for the automated detection of single nucleotide polymorphisms and small deletions or insertions.

Since the identification of the BRCA1 and BRCA2 genes >500 sequence variants have been detected world-wide. Approximately 60-70% were truncating mutations and the remainder were polymorphisms or missense mutations. It is clear for complete analysis of BRCA1 and BRCA2 that the protein truncation test (PTT) is not sufficient, as missense mutations do not alter the protein size and therefore cannot be analysed by PTT. As both genes are large, (21 and 27 exons respectively), with exon 11 in both genes accounting for almost 60% of coding regions, sequencing of the coding and flanking regions would be time consuming and expensive. The alternative method of dHPLC was chosen to produce a comprehensive analysis of BRCA1 and BRCA2 sequence variants in breast/ovarian cancer patients in the Irish population.

We have identified 2 BCRA1 mutations, 1294del40 and E143X, which appear to be common in the Irish population. Currently we have analyzed 25 exons from BRCA2 and 21 exons from BRCA1 in 116 patients, from a larger cohort of approximately 230 patients. Analysis is ongoing, but to date we have identified 85 BRCA2 sequence variants in 58 patients. Some of these appear to be common polymorphisms in the Irish population, others are non-conservative mutations or mutations of unkown effect.

© 2002 Cancer Research UK 

**P190**

**AP-1 AS A MEDIATOR OF ANDROGEN REFRACTORY PROSTATE CANCER**

*N Sarath Krishna[‡], J Edwards[‡], M A Underwood[‡], K M Grigor[†] & J M S Bartlett[‡]

[‡]University Department of Surgery & Urology, Glasgow Royal Infirmary & [†]Western General Hospital, Edinburgh.

**Introduction**-Hormonal therapy remains the treatment of choice for metastatic prostate cancer. However development of androgen refractory state is a serious clinical problem, for which there is currently no effective therapy. AP-1 is a complex of transcription factors c-Jun & c-fos, activated by Protein Kinase C. These proteins are up regulated in prostate cancer cell lines in the absence of androgens & there is a strong evidence that PKC activity is required for the survival and growth of androgen independent human prostate cancer cells. In-vivo studies are yet to be carried out. We propose to investigate this using human prostate cancer tissue.

**Aims**-1.To investigate the levels of expression of c-Jun, c-fos & AR proteins in paired pre & post cancer tissue from prostate cancer patients who have developed androgen refractory state.

2.To identify patients in whose tumours up regulation of AP-1 may be mediating androgen refractory state.

**Methods**-Forty-one patients with prostate cancer who have progressed to androgen refractory state were identified retrospectively from their clinical records. Their pre & post androgen refractory prostate tumour tissue [41 paired samples] provided material for study. Phosphorylated c-Jun, c-fos, AR & PSA levels of expression were quantified by immunohistochemistry.

**Results**-PSA was expressed in all the tissue samples tested indicating activation of androgen regulated genes. Increased expression of c-Jun/c-fos was observed in 32% [13/41] of post androgen refractory prostate cancer samples when compared with pre androgen refractory values. AR expression remained unaltered or decreased in 56% [23/41] of post androgen refractory prostate cancer biopsies when compared to the matched pre androgen refractory tissue. AR expression was increased in 44%. No significant difference in c-Jun/fos expression was observed in tumours with increased AR vs no change in AR expression.

**Conclusion**-We have demonstrated up-regulation and activation (via phosphorylation of c-Jun) of the AP-1 transcription factor in a significant subset [32%] of patients who have progressed to androgen refractory prostate cancer, independent of changes in AR expression. In this subset of patients, AP-1 may have a dominant effect in cancer progression via activation of androgen regulated genes by an AR independent mechanism. AP-1 could be a potential target for future therapies against androgen refractory prostate cancer in a selected group of patients.

**P191**

**ALTERATIONS IN MITOCHONDRIA AND MITOCHONDRIAL DNA-ENCODED ENZYMES IN RAT BLADDER TUMORS INDUCED BY BBN**

*Guang-fu Chen[1], Franky L Chan[2], Peter SF Chan[1]

Departments of Surgery[1] and Anatomy[2], Prince of Wales Hospital, Chinese University of Hong Kong, Hong Kong

**Objectives**

This study investigated the ultrastructural changes in mitochondria and changes in the mitochondrial DNA-encoded enzymes of oxidative phosphorylation in bladder cancer induced by BBN [N-butyl-N-(4-hydroxybutyl) nitrosamine] in rats.

**Methods**

A total of 180 male SD rats were given drinking water containing 0.05% BBN at libitum for 4 to 28 weeks. Control rats were given tap water without BBN. The normal bladder and tumor were processed for electron and light microscopy, and immunohistochemistry for mitochondrial enzymes of oxidative phosphorylation such as cytochrome *c* oxidase subunit II (COX II), cytochrome *c*, ubiquinol cytochrome *c* oxidoreductase, cytochrome *c* oxidase and ATP synthase. Protein expressions of mitochondrial enzymes were analyzed by Western blotting using the same antibodies for immunohistochemistry. The mRNA expression of mitochondrial enzymes was also analyzed by RT-PCR and Northern blotting. For RT-PCR analyses, normal and hyperplastic transitional epithelia were removed by laser capture microdissection.

**Results**

Both transitional cell carcinoma and squamous cell carcinoma were induced in the rat bladder after treatment with BBN for 16 to 28 weeks. Our results demonstrated a significant increase in the number of mitochondria in the BBN-induced carcinogenesis. Analyses of protein expressions of mitochondrial enzymes showed that these mitochondrial enzymes were increased in the BBN-induced bladder carcinomas. Similar increase in their mRNA expression levels was detected by RT-PCR. Northern blot analysis of COX II using a 218-bp RNA probe also showed a similar increase in mRNA levels of this enzyme.

**Conclusions**

Our study showed that there was significant increase in the number of mitochondria, and significant increases in both protein and mRNA expression of mitochondrial enzymes of oxidative phosphorylation in BBN-induced hyperplastic bladder epithelia and carcinomas as compared to normal bladder epithelium. These observations suggest that mitochondria and mitochondrial DNA-encoded enzymes might play an important role in BBN-induced rat bladder carcinogenesis.

**P192**

**FUNCTIONAL ANALYSIS OF THE RASSF1A TUMOUR SUPPRESSOR GENE USING YEAST TWO-HYBRID SCREEN**

S.L.Fenton*, L.B.Hesson, A.Agathanggelou, E.R. Maher, F Latif.

Dept of Medical and Molecular Genetics, Medical School, University of Birmingham, B15-2TT.

We and others have shown that multiple tumour suppressor genes may reside on chromosome 3p and that their inactivation plays a role in the pathogenesis of a number of common cancers including breast and lung. One newly identified gene located at 3p21.3 is the tumour suppressor gene RASSF1A. Previously, we demonstrated that inactivation of RASSF1A by allelic loss and methylation is a critical step in the development of a number of cancer types including breast, lung, neuroblastoma, and kidney tumours. The characterisation of proteins encoded by tumour suppressor genes such as RASSF1A, their binding partners and their location is crucial to understanding the function of a protein and its role in tumour development.

A yeast two-hybrid screen was performed in order to look for novel RASSF1A binding partners using MATCHMAKER system 3 (CLONTECH). The bait vector PGBKT7-RASSF1A (56-871bp) was constructed and transformed in yeast strain *S. cerevisiae* AH109 (MATa). The bait RASSF1A-BD was then used to screen a pretransformed MATCHMAKER brain library cloned in pACT2 and introduced into yeast strain *S.cerevisiae* Y187 (MATa). A total of 6 x $10^6$ clones were screened, of which 103 were positive for α-galactosidase expression. Clones were rescued and retransformed into yeast to reconfirm positive interactions. Positive clones were sequenced. From the above screen we identified ten independent clones, which encode for known genes involved in transcription regulation, cell division and cell signalling. In addition we also identified five novel genes in our screen of RASSF1A binding proteins.

We are now in the process of confirming all of our interacting clones *in vitro* using coimmunoprecipitation and GST fusion pull downs. The full length cDNAs of the interacting proteins and deletion constructs will also be studied using these techniques to identify interacting regions. We will then go on to verify these interactions *in vivo* in their native environments using mammalian MATCHMAKER Two-hybrid using the pCMV-Myc and pCMV-HA vectors and by transfecting into mammalian cell lines in order to observe the effects of *in vivo* expression on RASSF1A signalling. Clinically, this is extremely important because we need to establish the function of tumour suppressor genes such as RASSF1A if we are to restore normal gene function

© 2002 Cancer Research UK

### P193

### RNA POLYMERASE III TRANSCRIPTION CAN BE DEREPRESSED BY ONCOGENES OR MUTATIONS THAT COMPROMISE p53 FUNCTION IN TUMOURS AND LI-FRAUMENI SYNDROME

Torsten Stein[1], Diane Crighton[1*], Jennifer Morton[1], John M. Boyle[2], Jennifer M. Varley[2] and Robert J. White[1]

[1]Institute of Biomedical and Life Sciences, Division of Biochemistry and Molecular Biology, Davidson Building, University of Glasgow, Glasgow, G12 8QQ, U.K.

[2]Cancer Research Campaign Department of Cancer Genetics, Paterson Institute for Cancer Research, Wilmslow Road, Manchester, M20 9BX, U.K.

RNA polymerase (pol) III synthesizes essential small RNAs, including tRNA and 5S rRNA. Wild-type p53 can repress pol III transcription both in vitro and in vivo (1-2). Many tumours carry substitutions in p53 which have selective effects on its functions. We identify tumour-derived mutations that compromise the ability of p53 to regulate pol III transcription (3). Furthermore, substitution R175H, the most common mutation in cancers, converts p53 from a repressor to an activator of pol III. Human papillomavirus E6 and cellular hdm2 can both release pol III from repression by p53. These data suggest that the restraining influence of p53 on pol III will be lost in many tumours. In addition to these features of sporadic cancers, some individuals inherit mutant forms of p53 and consequently suffer from Li-Fraumeni syndrome, showing genetic predisposition to certain malignancies. We find that pol III transcriptional activity is often highly elevated in primary fibroblasts from Li-Fraumeni patients, especially if the germline p53 mutation is followed by loss of the remaining allele. Our data suggest that p53 status can have a profound effect upon pol III transcription and hence on the biosynthetic capacity of cells.

1. Chesnokov, I., Chu, W.-M., Botchan, M. R. and Schmid, C. W. (1996) Mol Cell Biol 16, 7084.
2. Cairns, C. A. and White, R. J. (1998) EMBO J. 17, 3112.
3. Stein, T., Crighton, D., Boyle, J. M., Varley, J. M. and White, R. J. (2002) Oncogene In press.

### P194

### IDENTIFICATION OF A USF INITIATOR / ACTIVATOR REGION OF THE VASOPRESSIN PROXIMAL PROMOTER IN LUNG CANCER

Jodie L. Edgson[1*]; Judy M. Coulson[2]; Penella J. Woll[1].
[1]Cancer Research UK, Academic Unit of Clinical Oncology, University of Nottingham, UK. [2]Physiological Laboratory & Human Anatomy and Cell Biology, University of Liverpool, UK.

Arginine vasopressin (AVP) is a neuropeptide that is expressed in small cell lung cancer (SCLC) but not in non small cell lung cancer (NSCLC). On the over expression of the transcription factors Upstream Stimulatory Factor 1 and/ or 2 (USF-1 and USF-2), the vasopressin promoter can be activated and studied in NSCLC. Here we investigate the motif through which these transcription factors activate.

**Methods**: PCR mediated site-directed mutagenesis was carried out within the promoter region to generate mutations at three sites, the C-Ebox, E-Motif and the TATAbox. The fragments were initially cloned into the pCR2.1 vector then subcloned into chloramphenicol acetyl transferase (CAT) reporter constructs. Electroporation was used as a transfection method to introduce the reporter constructs into NCI-H460, a NSCLC cell line. Co-transfection was with a Beta-Galactosidase reporter gene construct to standardise the individual experiments. Reporter gene assays were used to measure the level of promoter activity.

**Results**: The mutations were generated within two different sized fragments: (-52 to +42) and (-52 to +56), the larger promoter fragments also included a mutated D-Ebox. The assays showed the same pattern of activity for the two sets of promoter constructs when cotransfected with the USF-2 transcription factor. The effect of USF-1 on the constructs (-52 to +42) was similar to that of USF-2 but at a reduced level. The mutations at the C-Ebox had no effect on promoter activity. In contrast mutations at the E-Motif and those with double mutations at both the E-Motif and C-Ebox abolished activity of the promoter. Mutations at the TATAbox also resulted in minimal activity of the promoter.

**Discussion**: The E-Motif and the TATAbox were both required for activation of the vasopressin promoter in NSCLC. However it was clear that the E-Motif is the promoter site through which USF activation of AVP is mediated in lung cancer.

### P195

### TUMOUR NECROSIS FACTOR-α CELL SIGNALLING EVENTS IN HUMAN MESOTHELIAL CELLS.

Swain W[1]*, Houghton C[1], Coyle E[1], Edwards JG[2], Faux S[1], Gant T[1], O'Byrne KJ[2]. [1]MRC Toxicology Unit and [2]Department of Oncology, University of Leicester, LE1 5WW.

Recent experimental evidence indicates that chronic immune activation, associated with angiogenesis, suppression of cell mediated immunity and inhibition of apoptosis, plays a central role in the development of malignant disease. Malignant mesothelioma (MM) represents a pleural tumour which fits this model. Crocidolite fibres are intimately associated with the development of MM. These fibres have been demonstrated to upregulate epidermal growth factor receptor (EGFR) expression and to induce cell signalling events, including activation of the phosphotidylinositol-3-kinase/Akt and MAP-kinase pathways in human mesothelial cells. Following inhalation, carcinogenic asbestos fibres also induce the release of a number of cytokines from pulmonary macrophages including tumour necrosis factor (TNF)-α. We examined the effects of TNF-α exposure on simian virus-40 immortalised human mesothelial Met5A cells. TNF-α induced upregulation and phosphorylation of the EGFR. This was blocked by pre-treatment with N-acetyl-cysteine indicating that the effects were due to oxidative stress. Using gel mobility shift assays TNF-α increased transcription factor NF-κB binding to DNA, an effect partially blocked by pre-treatment with the selective EGFR tyrosine kinase inhibitor PKI166. TNF-α exposure was associated with increased cell proliferation, an effect completely abrogated by exposure to the NF-κB decoy peptide SN50. Gene microarray analysis revealed upregulation of a range of genes within the Met5A cells including the apoptosis inhibitors CIAP1 and 2 and FLIP and the pro-angiogenic cytokines IL-6 and IL-8 implicated in the suppression of cell mediated immune responses. These results indicate that TNF-α induces EGFR cell signalling events which contribute to the activation of NF-κB by the cytokine. Activation of NF-κB appears crucial to TNF-α induced cell proliferation. Furthermore TNF-α exposure results in the upregulation of genes capable of inducing a local chronic immune activation state which would favour the survival and subsequent mutation of stress induced transformed pre-malignant cells. The findings suggest that TNF-α plays a central role in MM tumourigenesis.

### P196

### INTERLEUKIN-8 (IL-8) PROMOTES AUTOCRINE GROWTH OF PROSTATE CANCER CELLS

Maryalice Hamill*, Johanna Messenger, Monica Monaghan, Jayne Draffin, Patrick Johnston and David Waugh
Department of Oncology, Queen's University Belfast, Belfast City Hospital, Belfast, BT9 7AB

Prostate cancer is currently the most prevalent cancer in men and the second leading cause of cancer-related deaths in males. Over-expression of the chemokine IL-8 has been reported in the sera of patients with localized and metastatic cancer of the prostate. Animal models have also positively correlated the expression of IL-8 with the tumorigenicity and metastasis of prostate cancer. However, the underlying mechanism by which IL-8 appears to promote disease progression remains poorly understood. Since IL-8 has been proposed to act as an autocrine growth factor in melanoma and colon cancer, we initiated a study to determine the effect of IL-8 upon the growth of two prostate cell lines, the metastatic PC3 cell line and the transformed PNT1A epithelial cell line. IL-8 administration produced a consistent, concentration-dependent increase in PC3 cell growth as assessed by cell count analysis. The response had an apparent EC50 of 1nM and produced a mean increase of 45% in cell number when compared to untreated cells. The PNT1A cell line demonstrated negligible response to exogenous IL-8. Expression of the IL-8 receptors, CXCR1 and CXCR2, was detectable in both cell lines using RT-PCR. Therefore, further analysis of receptor

© 2002 Cancer Research UK

